# Partner valves: role of a prospective device selection strategy using ACURATE and SAPIEN 3 as complementary devices in transcatheter aortic valve implantation

**DOI:** 10.3389/fcvm.2026.1710186

**Published:** 2026-05-27

**Authors:** Jonathan M. Michel, Mi Chen, Neria E. Winkler, Philipp Theil, Jan A. Kleeberger, Victor Schweiger, Alessandro Candreva, Alexander Gotschy, Julia Stehli, Bernhard Haubner, Thierry G. Donati, Janina Kowalik, Dijana Savic, Christian Templin, Barbara E. Stähli, Felix C. Tanner, Thomas Gilhofer, Albert M. Kasel

**Affiliations:** 1 Department of Cardiology, University Heart Center, University Hospital Zurich, Zurich, Switzerland; 2 Faculty of Medicine, University of Zurich, Zurich, Switzerland; 3 Department of Cardiology, Angiology and Intensive Care Medicine, Deutsches Herzzentrum der Charité (DHZC), Berlin, Germany; 4 Faculty of Medicine, Medical University of Gdansk, Gdańsk, Poland; 5 Department of Cardiology, Greifswald University Hospital, Greifswald, Germany; 6 Department of Cardiology, Winterthur Hospital, Winterthur, Switzerland

**Keywords:** aortic stenosis, balloon-expandable, clinical outcomes, self-expanding, TAVI, TAVR

## Abstract

**Background:**

Transcatheter aortic valve implantation (TAVI) outcomes using balloon-expandable valves (BEV) and self-expanding valves (SEV) differ owing to device-specific technical features and anatomical factors.

**Objectives:**

This study aimed to evaluate prospective TAVI device selection based on anatomic features to optimise outcomes compared with published randomised data using two newer-generation devices.

**Methods:**

A total of 607 consecutive patients with severe aortic stenosis treated at a single centre from 2020 to 2023 underwent TAVI using a prospective device selection strategy. Heavy and/or asymmetric valve calcification and bicuspid anatomy were preferentially treated with SAPIEN 3/SAPIEN 3 ULTRA BEV, whereas small annulus dimensions and less calcified valves were preferentially treated with ACURATE neo/ACURATE neo2 SEV. In-hospital Valve Academic Research Consortium-3 endpoints and 1-year mortality were evaluated.

**Results:**

ACURATE was used in 182 (30%) patients and SAPIEN 3 in 425 (70%) patients. The proportion of women was higher in the ACURATE group (65% vs. 31%, *p* < 0.001), and patients were older (84 vs. 80 years, *p* < 0.001). Technical success (97% vs. 97%, *p* > 0.99) and new permanent pacemaker implantation during index hospitalisation (10% vs. 10%, *p* > 0.99) did not differ between the ACURATE and SAPIEN 3 groups. Intended performance was higher in the ACURATE group than in the SAPIEN 3 group (98% vs. 91%, *p* = 0.005), with aortic regurgitation ≥moderate (2% vs. <1%, *p* = 0.32), and mean transvalvular gradient ≥20 mmHg (<1% vs. 4%, *p* = 0.043). The estimated mortality at 1 year was 14% versus 12% (log-rank *p* = 0.60) in the ACURATE and SAPIEN 3 groups, respectively.

**Conclusions:**

A prospective device selection strategy using two complementary TAVI valves resulted in comparable outcomes between the SEV and BEV devices. In-hospital intended performance was higher in the ACURATE group. Despite baseline group differences, there was no difference in 1-year mortality.

## 1 Introduction

Transcatheter aortic valve implantation (TAVI) is approved for treating severe aortic stenosis (AS) in all surgical-risk groups. Despite a reduction in periprocedural adverse events with increased operator experience and iterative improvements in transcatheter heart valve (THV) technology, vascular complications, stroke, paravalvular leak (PVL), and new permanent pacemaker implantation (PPI) remain the most clinically significant and undesirable device-specific complications (1). THV devices can be broadly divided into balloon-expandable valve (BEV) and self-expanding valve (SEV) categories, and real-world registry data indicate differences in periprocedural adverse outcomes between BEV and SEV devices, particularly higher rates of PVL and PPI in patients treated with SEV (2). Conversely, BEV use is associated with higher transvalvular gradients, although the prognostic impact remains uncertain, and higher rates of annulus injury (3). Recent randomised trial data in patients with a small aortic annulus also demonstrated the favourable transvalvular gradient advantage of SEV compared with BEV (4).

The ACURATE neo™ and latest-generation ACURATE neo2^TM^ SEV (Boston Scientific, Marlborough, MA, USA) received regulatory approval in Europe in September 2014 and April 2020, respectively. The ACURATE device has a supra-annular design with a comparatively lower radial force at the annulus level. Compared with the two leading TAVI devices in randomised head-to-head studies, the ACURATE neo did not meet the criteria for non-inferiority, in part due to higher rates of PVL; however, the rate of new PPI was comparable with the SAPIEN 3 BEV and lower than the Evolut SEV, and transvalvular gradients were lower than those with the SAPIEN 3 device (5, 6). Published haemodynamic data for the ACURATE and SAPIEN 3 platforms are summarised in Table 1. Although SAPIEN 3 has a high radial force with low reported PVL and lower new PPI (7, 8), ACURATE has a comparatively moderate radial force and lower gradient in small anatomy with lower new PPI (5).

**Table 1:** Existing randomised study haemodynamic outcomes at 30-days and 1-year mortality using ACURATE neo/ACURATE neo2 and SAPIEN 3/ Ultra THV.

Study	Comparison groups	Key exclusion criteria	Sample size	30 days			1 year
				≥Moderate AR (%)	Mean gradient mmHg	Mean gradient ≥20 mmHg (%)	All-cause mortality (%)
Lanz et al. (2019) (5) SCOPE I	ACURATE neo vs. SAPIEN 3	Bicuspid valve, emergency procedure, LVEF <20%	ACURATE neo *n* = 372	9.6	7 (5–9)	1.2	10
			SAPIEN 3 *n* = 367	2.8	11 (8–14)	NR	8
Mack et al. (2019) (8) PARTNER 3	SAPIEN 3 vs. SAVR in low-risk patients	Bicuspid valve, emergency procedure, LVEF <30%	SAPIEN 3 *n* = 496	0.8	12.8 ± 0.2	NR	1.0
Tamburino et al. (2020) (6) SCOPE II	ACURATE neo vs. CoreValve Evolut	Bicuspid valve, LVEF <20%	ACURATE neo *n* = 398	9.6	6.3 ± 2.8 *n* = 257	NR	13
Herrmann et al. (2024) (4) SMART	SAPIEN 3/3 Ultra vs. Evolut	Annulus area ≥430 mm^2^, bicuspid valve, emergency procedure, LVEF <20%	SAPIEN 3/3 Ultra *n* = 371	0.9	NR^a^	9.7^b^ *n* = 361	5.9
Makkar et al. (2025) (9) ACURATE IDE	ACURATE neo2 vs. SAPIEN 3/EVOLUT	Bicuspid valve, LVEF <20%	ACURATE neo2 *n* = 752	3.4	8 ± 3 *n* = 679	NR	5.0
			SAPIEN 3/3 Ultra *n* = 504	0.2	12 ± 5 *n* = 462	NR	4.1

Values are presented as median (interquartile range) or mean ± SD. THV, transcatheter heart valve; AR, aortic regurgitation; LVEF, left ventricular ejection fraction; NR, not reported.

a Mean gradient reported at 12 months = 15.7 ± 6.7 mmHg, *n* = 365.

b Mean gradient ≥20 mmHg or Vmax ≥3 m/s.

The ACURATE neo2 was subsequently evaluated for approval by the United States Food and Drug Administration (FDA) in the ACURATE IDE study (Trial ID: NCT03735667). The study failed to demonstrate the non-inferiority of the ACURATE neo2 compared with commercially available valves (SAPIEN 3/3 Ultra and Evolut Platforms) regarding the combined endpoint of 1-year death, stroke, or rehospitalisation (16.2% in the ACURATE neo2 group vs. 9.5% in the conventional TAVI group; between-group difference = 6.6%, non-inferiority margin = 8.0%, and posterior probability of treatment difference >0.999) (9). During the study, 72% of operators implanted five or fewer devices, and, in *post hoc* analysis, approximately 20% of patients received an underexpanded ACURATE THV; there was a higher rate of the primary outcome among this group compared with those with a well-expanded valve (18.8% vs. 12.4%, respectively, *p* = 0.05). Subsequent non-randomised data from Europe showed similar 1-year outcomes when ACURATE was compared with the SAPIEN 3 device (10). Shortly before the completion of the current analysis, Boston Scientific withdrew the ACURATE platform from the worldwide market, citing excessive financial burdens associated with maintaining and obtaining regulatory approval (11).

The previously reported differences in outcomes between the ACURATE and SAPIEN 3 devices may have been due to suboptimal patient selection and device deployment. We hypothesised that an all-comer group of patients with severe native valve AS could be treated with either the ACURATE or SAPIEN 3 platform, achieving improved haemodynamic outcomes compared with existing randomised trial data, using a prospective device selection strategy.

## 2 Methods

### 2.1 Study population

**Figure 1:**
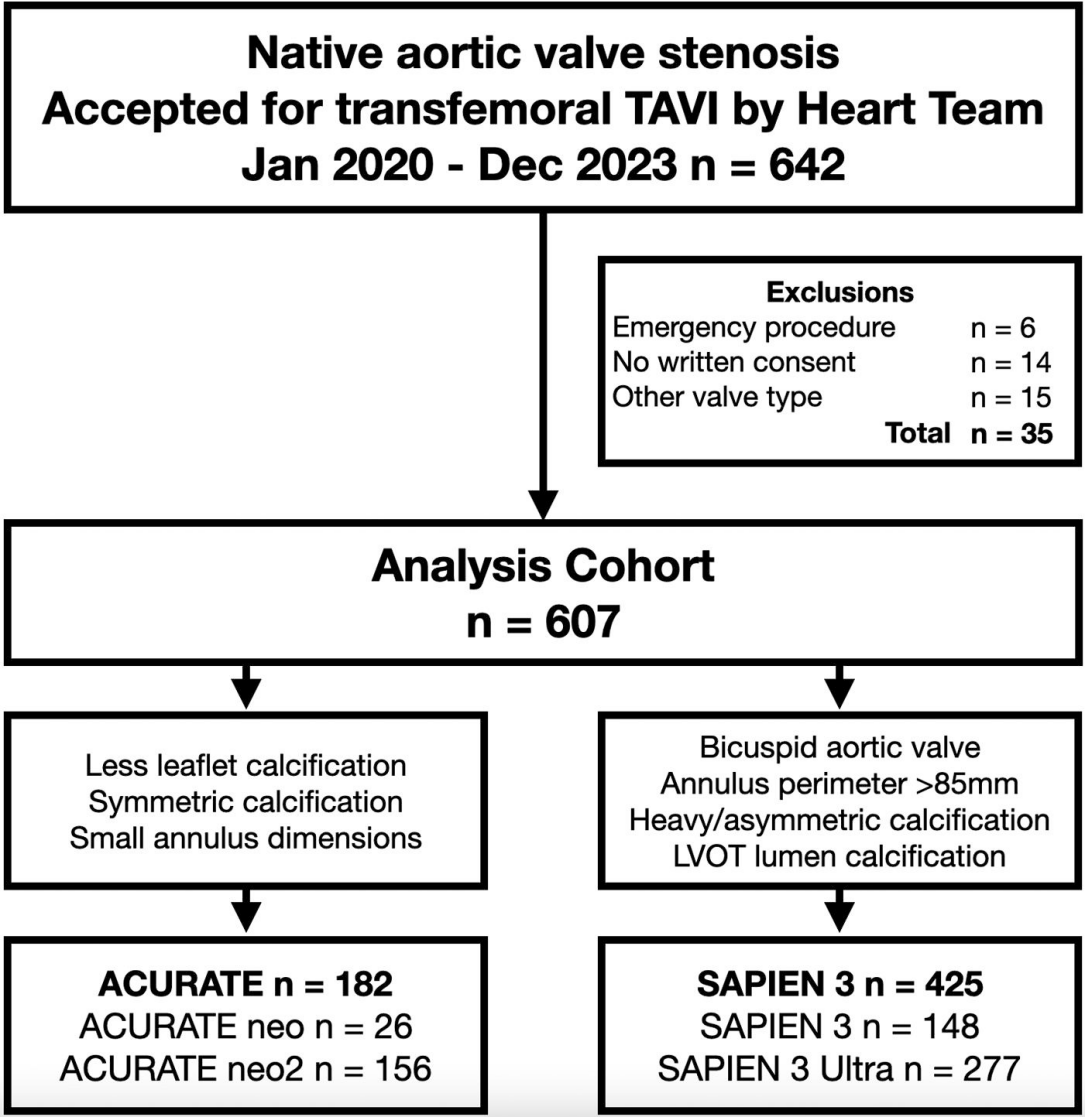
Patient enrolment flow diagram. During the study period, 15 patients received alternative THV (Medtronic Evolut Pro/R in 13 cases and Abbott Navitor in 2 cases), The reasons for alternative device use included coronary obstruction risk–retrievable device required (*n* = 5), small annulus with excessive calcification for ACURATE (*n* = 1), device availability/logistical factors (*n* = 4), device selection by external operator (*n* = 2), and testing of novel device (*n* = 2). Six patients who underwent emergency TAVI were excluded because of the very high associated mortality risk.

All patients treated with TAVI for severe native valve AS between January 2020 and December 2023 receiving a Boston Scientific self-expanding (ACURATE neo™ or ACURATE neo2™) or Edwards Lifesciences balloon-expandable (SAPIEN 3™ or SAPIEN 3 Ultra™) THV via the transfemoral approach in the Department of Cardiology, University Hospital Zurich, Zurich, Switzerland, were included in this prospective registry study (ACURATE and SAPIEN 3 groups, respectively). The indication for TAVI was confirmed by a multidisciplinary heart team. All patients provided ethically approved written informed consent for data collection and their evaluation. Patients who underwent emergency procedures, had unavailable written consent, or underwent other valve types (6%, 35/642) were excluded (Figure 1). A subgroup analysis was performed on patients with an annulus area of <430 mm^2^.

### 2.2 Echocardiography and multislice computed tomography analysis

The preoperative evaluation included non-contrast and contrast-enhanced multislice computed tomography (MSCT) with multi-planar reconstruction and measurement of the aortic annulus and contrast-enhanced scan-specific aortic valve calcium volume, as previously described (12). The Agatston Score was determined using non-contrast images. Transthoracic echocardiography was performed before TAVI and before discharge from the hospital.

### 2.3 Device selection and procedure

We hypothesised that an all-comer TAVI cohort could be treated using a combination of the ACURATE SEV and SAPIEN 3 BEV platforms and achieve improved clinical and haemodynamic outcomes compared with existing randomised data. The ACURATE and SAPIEN 3 platforms were selected specifically for this prospective analysis because of the similarities between the low-profile 14F hydrophilic expandable delivery sheath (16F for the larger 29 mm BEV), the flexible delivery system, the comparatively low rate of new PPI, the sealing skirt to minimise PVL (ACURATE neo2 and SAPIEN 3, with an improved skirt on the SAPIEN 3 Ultra), and the favourable stent frame design facilitating subsequent coronary access; these features were deemed conducive to a minimalist transfemoral TAVI approach. All patients were treated in a standardised minimalist setting with conscious sedation (except pre-intubated patients), fluoroscopy guidance, and, whenever feasible, immediate removal of the femorally placed temporary pacemaker, as previously described (12).

The SAPIEN 3 device was considered the default THV of choice, with selected patients receiving the ACURATE device based on anatomic features. Heavy and/or asymmetric calcification of the aortic valve leaflets and annulus, or calcification protruding into the left ventricular outflow tract (LVOT) lumen, was treated with the higher radial force SAPIEN 3 device to reduce the risk of PVL. The SAPIEN 3 device was also preferred in younger patients (the low frame height may facilitate subsequent valve-in-valve TAVI) and in those with challenging femoral access (robust hydrophilic sheath). Notably, owing to ease-of-use, SAPIEN 3 was strongly favoured in critically ill patients requiring emergency TAVI, and the ACURATE device did not have regulatory approval for use in valve-in-valve procedures; hence, these patients were excluded from this study. Less calcified aortic valves were preferred for the ACURATE device, as were smaller annuli (mean diameter, ≤21 mm) to reduce the risk of an elevated transvalvular gradient in small anatomy.

THV type and size selection was performed independently—using the strategy described above—before the procedure by two experienced structural interventionalists (AK and JM), and in case of initial disagreement, consensus was reached. All patients in the ACURATE group underwent predilatation according to the instructions for use. Large-bore access site preclosure was performed using the Perclose ProGlide™/ProStyle™ (Abbot, Chicago, IL, USA) suture device. Embolic protection devices were not routinely employed.

### 2.4 Endpoints

In-hospital clinical outcomes were obtained from medical records. The clinical and composite endpoints were consistent with the Valve Academic Research Consortium-3 (VARC-3) criteria (13). In summary, technical success was defined as freedom from mortality, successful access, delivery, and correct positioning of a single device, retrieval of the delivery system, and freedom from surgery or intervention related to the device or to a major vascular or access-related or cardiac structural complication. Device success was defined as technical success, freedom from mortality, freedom from surgery or intervention related to the device or to a major vascular or access-related or cardiac structural complication, and intended performance of the valve [mean gradient <20 mmHg, peak velocity <3 m/s, Doppler velocity index ≥0.25, and less than moderate aortic regurgitation (AR)]. Follow-up data were available for 100% of patients at discharge, and 1-year survival status (±2 months) was available for 96% of patients.

### 2.5 Statistical analysis

Categorical variables were summarised as counts and percentages and compared using Pearson's *χ^2^* or Fisher's exact test, as appropriate. The distribution of continuous variables was assessed using the Shapiro–Wilk test, reported as mean ± standard deviation or median and interquartile range, and compared using the *t*-test or Mann–Whitney–Wilcoxon *U* test, respectively. A Kaplan–Meier analysis was performed to evaluate the survival distribution at 1-year between groups, using the log-rank test to examine the equality of the survival distributions. Univariate and multivariate linear regression analyses were used to evaluate the association between factors potentially relevant to the aortic valve effective orifice area [gender, left ventricular ejection fraction (LVEF) <30%, predilatation, postdilatation, THV type, age, EuroSCORE II, aortic annulus area, and Agatston score] and postprocedural indexed effective orifice area (iEOA). R Software (version 4.1.2) was used for statistical analysis. Significance was set at a two-sided alpha level of 0.05.

## 3 Results

**Figure 2:**
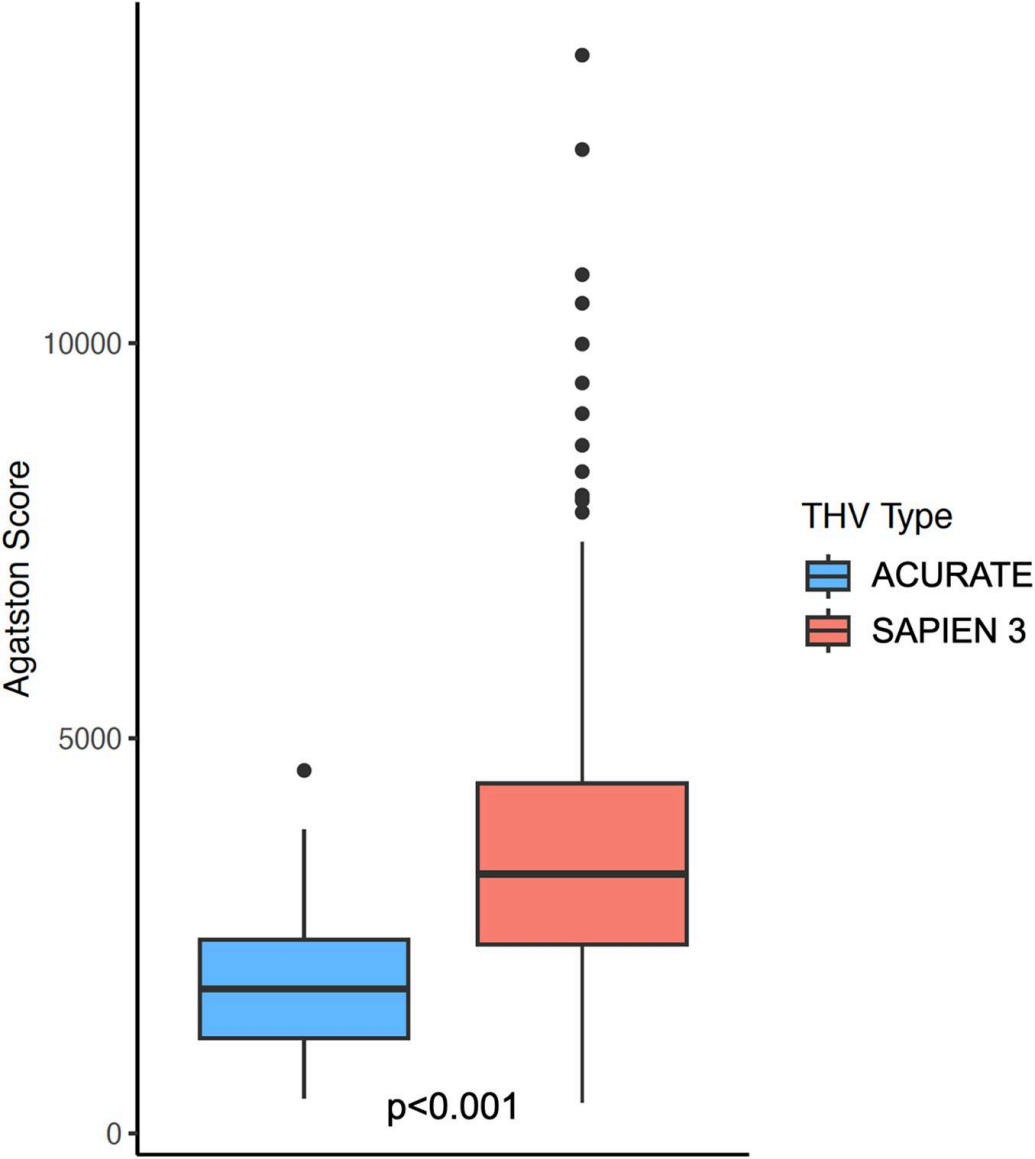
Aortic valve Agatston score by THV type. Box and whisker plot displaying median, interquartile range, and minimum and maximum aortic valve Agatston scores with outliers for all patients with available data (ACURATE, *n* = 162; SAPIEN 3, *n* = 390). THV, transcatheter heart valve.

**Figure 3:**
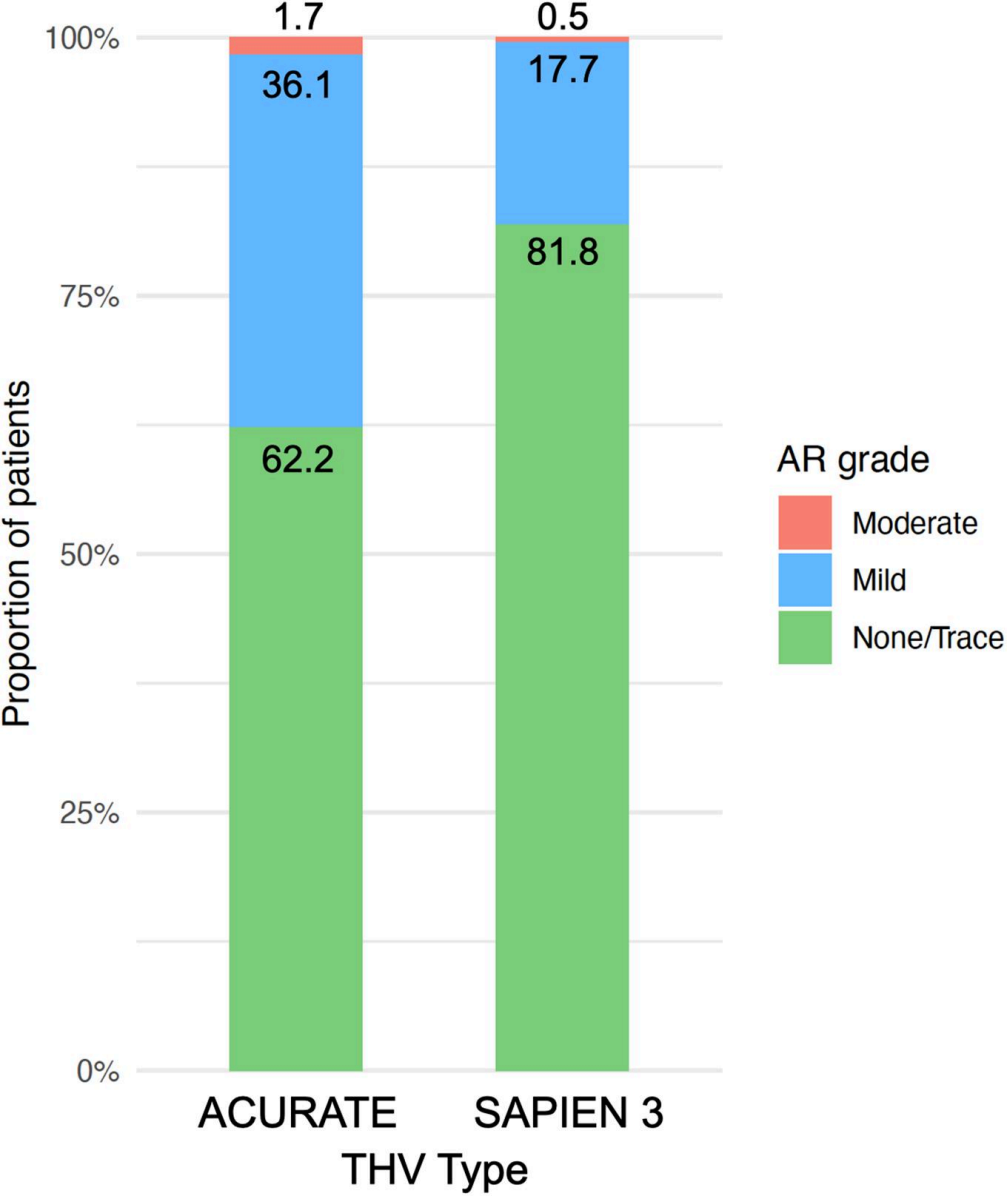
Postprocedural in-hospital aortic regurgitation grade in transthoracic echocardiography. AR, aortic regurgitation; THV, transcatheter heart valve.

The baseline clinical and aortic valve characteristics are shown in Table 2. The median age was 81 (76–86) years, and 41% of the patients were women, with a median EuroSCORE II of 4.9% (2.8%–9.5%). An ACURATE device was implanted in 30% (182/607) of patients [14% ACURATE neo (26/182) and 86% ACURATE neo2 (156/182)], and a SAPIEN 3 device was implanted in 70% (425/607) of cases [35% SAPIEN 3 (148/425) and 65% SAPIEN 3 ULTRA (277/425)]. Patients receiving ACURATE were significantly older, with a higher EuroSCORE II and a higher proportion of women. As a result of prospective device selection, patients treated with SAPIEN 3 had larger annulus dimensions, a higher rate of bicuspid valves, and qualitatively and quantitatively more severe valve calcification, including a higher Agatston score (Figure 2). Table 3 shows the procedural characteristics and in-hospital outcomes. The procedure was completed under conscious sedation in 98% of the cases. Arterial disease (a composite of peripheral arterial, carotid, and aortic disease) was more frequent in the ACURATE group; nevertheless, the use of a super-stiff wire (Meier, Boston Scientific) for primary vascular sheath insertion was more common in the SAPIEN 3 group. Technical success was achieved in 97% of the patients in both groups (*p* > 0.99). Predilatation was performed in 100% of ACURATE cases compared to 46% of SAPIEN 3 cases; in the ACURATE group, the diameter of the predilatation balloon was within 2.0 mm of the mean annulus diameter in 97% of patients. The transvalvular gradient was lower in the ACURATE group (7.2 ± 3.0 vs. 11.1 ± 4.1, *p* < 0.001), resulting in an intended performance of 98% in ACURATE and 91% in SAPIEN 3 patients (*p* = 0.005). Elevated mean transvalvular gradient ≥20 mmHg was observed in <1% and 4% (*p* = 0.021) of the patients in the ACURATE and SAPIEN 3 groups, respectively. The rates of postprocedural AR were low in both groups (Figure 3), with no significant difference in the rate of moderate AR; 2% versus <1% (*p* = 0.32) in the ACURATE and SAPIEN 3 groups, respectively. None of the 26 patients treated with the older ACURATE neo device had ≥moderate aortic regurgitation before discharge. No patient had postprocedural AR greater than moderate.

**Table 2:** Baseline clinical and aortic valve characteristics.

Variable	Total *n* = 607	ACURATE *n* = 182	SAPIEN 3 *n* = 425	*p* value
Clinical characteristics				
Women	250 (41.2)	119 (65.4)	131 (30.8)	<0.001
Age (years.)	81 (76–86)	84 (79–88)	80 (75–85)	<0.001
EuroSCORE II (%)	4.9 (2.8–9.5)	5.8 (3.5–10.5)	4.6 (2.6–8.9)	0.001
BMI (kg/m^2^)	26.3 (23.5–29.9)	26.0 (22.3–30.4)	26.4 (24.1–29.8)	0.13
Hypertension	583 (96.4)	176 (96.7)	407 (96.2)	0.96
Hypercholesterolemia	508 (84.0)	144 (79.1)	364 (86.1)	0.044
Diabetes mellitus	216 (35.7)	67 (36.8)	149 (35.2)	0.78
eGFR (mL/min)	57 (43–74)	53 (40–67)	59 (44–76)	0.002
Chronic lung disease	90 (14.9)	26 (14.3)	64 (15.1)	0.89
Coronary artery disease	375 (62.0)	117 (64.3)	258 (61.0)	0.50
Previous MI	99 (16.4%)	29 (15.9)	70 (16.5)	0.95
Previous PCI	154 (25.5)	50 (27.5)	104 (24.6)	0.52
Previous cardiac surgery	47 (7.8)	17 (9.3)	30 (7.1)	0.43
Arterial disease	132 (21.8)	50 (27.5)	82 (19.4)	0.036
Cerebrovascular disease	73 (12.1)	26 (14.3)	47 (11.1)	0.34
LVEF (%)	56 (46–61)	56 (46–61)	56 (45–62)	0.47
Impaired LV function (LVEF <30%)	48 (7.9)	9 (4.9)	39 (9.2)	0.11
Previous pacemaker	63 (10.4)	23 (12.6)	40 (9.4)	0.29
Valve characteristics				
Transthoracic echocardiography				
Mean gradient (mmHg)	40 (29–46)	32 (24–41)	42 (33–49)	<0.001
Valve area—VTI (cm^2^)	0.8 (0.6–0.9)	0.8 (0.6–0.9)	0.8 (0.6–0.9)	0.90
Annulus dimensions				
Mean diameter (mm)	24.9 (23.3–26.5)	23.5 (22.5–24.9)	25.6 (24.1–27.0)	<0.001
Area (mm^2^)	480 (417–542)	429 (386–482)	505 (444–562)	<0.001
Perimeter (mm)	79 (74–84)	74 (71–79)	81 (76–85)	<0.001
Bicuspid aortic valve	93 (14.9)	2 (0.6)	91 (21.0)	<0.001
≥Moderate aortic regurgitation	54 (8.9)	12 (6.6)	42 (9.9)	0.25
Agatston score, *n* = 552	2,730 (1,956–3,836)	1,828 (1,208–2,453)	3,286 (2,388–4,430)	<0.001
Corrected for annulus area	5.70 (4.20–7.73)	4.21 (3.11–5.49)	6.43 (5.02–8.85)	<0.001
Qualitative valve calcification severity, *n* = 601^a^				<0.001
Mild	87 (14.5)	67 (37.2)	20 (4.8)	
Mild—moderate	133 (22.1)	66 (36.7)	67 (15.9)	
Moderate	184 (30.6)	40 (22.2)	144 (34.2)	
Moderate—severe	133 (22.1)	7 (3.9)	126 (29.9)	
Severe	64 (10.6)	0 (0.0)	64 (15.2)	
Asymmetric calcification	141 (23.5)	11 (6.1)	130 (30.9)	<0.001
Quantitative calcification volume (mm^3^), *n* = 586				
Total score	659 (406–1,068)	364 (221–523)	854 (553–1,219)	<0.001
Corrected for annulus area (mm^3^/mm^2^)	1.39 (0.87–2.05)	0.82 (0.54–1.20)	1.70 (1.14–2.51)	<0.001
Aortic valve	627 (382–1,024)	356 (219–471)	812 (522–1,174)	<0.001
LVOT	0 (0–45)	0 (0–13)	8 (0–63)	<0.001

Values are presented as median (interquartile range) or absolute number (percentage). BMI, body mass index; eGFR, estimated glomerular filtration rate; MI, myocardial infarction; PCI, percutaneous coronary intervention; LVEF, left ventricular ejection fraction; VTI, velocity time integral; LVOT, left ventricular outflow tract.

a CT artefact precluded calcification estimation in six patients.

**Table 3:** Procedural characteristics, in-hospital outcomes, and 30-day valve haemodynamic function.

Variable	Total *n* = 607	ACURATE *n* = 182	SAPIEN 3 *n* = 425	*p* value
Intubation				0.23
Elective	4 (0.7)	2 (1.1)	2 (0.5)	
Emergency	5 (0.8)	0 (0.0)	5 (1.2)	
Embolic protection device	2 (0.3)	0 (0.0)	2 (0.5)	0.88
Access vessel stiff wire				<0.001
None	4 (0.7)	2 (1.1)	2 (0.5)	
Amplatz extra stiff	313 (51.7)	24 (13.3)	289 (68.2)	
Safari	98 (16.2)	92 (50.8)	6 (1.4)	
Supra Core	20 (3.3)	20 (11.0)	0 (0.0)	
Meier	167 (27.6)	42 (23.2)	125 (29.5)	
Bilateral Meier	3 (0.5)	1 (0.6)	2 (0.5)	
Predilatation	376 (61.9)	182 (100.0)	194 (45.6)	<0.001
Predilatation within 2 mm of the mean annulus diameter		173 (96.6)	95 (50.8)	<0.001
Postdilatation	130 (21.4)	62 (34.1)	68 (16.0)	<0.001
Procedure time (min)	44 (37–52)	47 (41–56)	43 (35–50)	<0.001
Contrast (mL)	106 (88–130)	120 (99–144)	100 (86–121)	<0.001
Fluoroscopy time (min)	9.0 (6.6–11.5)	9.8 (7.9–12.1)	8.5 (6.3–11.1)	<0.001
THV implant size (mm)				
20		—	7 (1.6)	
23		37 (20.3)	76 (17.9)	
25		77 (42.3)	—	
26		—	202 (47.5)	
27		68 (37.4)	—	
29		—	140 (32.9)	
Technical success^a^	587 (96.7)	176 (96.7)	411 (96.7)	>0.99
Procedural death	0 (0.0)	0 (0.0)	0 (0.0)	NA
Successful access, retrieval, and deployment	607 (100.0)	182 (100.0)	425 (100.0)	NA
Correct position	606 (99.8)	182 (100.0)	424 (99.8)	>0.99
Freedom from surgery or intervention^b^	596 (98.2)	178 (97.8)	418 (98.4)	0.89
Device success,^c^ *n* = 595	541 (90.9)	171 (95.5)	370 (88.9)	0.016
Intended performance,^d^ *n* = 570	528 (92.6)	165 (97.6)	363 (90.5)	0.005
AR grade ≥ II	5 (0.8)	3 (1.7)	2 (0.5)	0.32
Elevated gradient (≥20mmHg)	18 (3.0)	1 (0.6)	17 (4.0)	0.043
Mean transvalvular gradient (mmHg)	10.0 ± 4.2	7.2 ± 3.0	11.1 ± 4.1	<0.001
Patient prosthesis mismatch^e^	138 (24.1)	25 (14.8)	113 (28.0)	0.001
In-hospital outcomes				
Days on IMC/ICU	1 [1, 1]	1 [1, 1]	1 [1, 1]	0.93
Length of stay	4 [3, 8]	5 [3, 8]	4 [3, 8]	0.27
All-cause mortality	11 (1.8)	3 (1.6)	8 (1.9)	>0.99
All stroke	23 (3.8)	8 (4.4)	15 (3.5)	0.78
Fatal/disabling stroke	12 (2.0)	3 (1.6)	9 (2.1)	0.95
Major vascular complication	41 (6.8)	15 (8.2)	26 (6.1)	0.44
Annulus rupture	2 (0.3)	0 (0.0)	2 (0.5)	0.88
Access site complication requiring intervention^f^	16 (2.6)	7 (3.8)	9 (2.1)	0.35
Bleeding type 2–4	30 (4.9)	12 (6.6)	18 (4.2)	0.31
Acute kidney injury grade 3	5 (0.8)	0 (0.0)	5 (1.2)	0.33
MI	2 (0.3)	0 (0.0)	2 (0.5)	0.88
New pacemaker^g^	54 (9.9)	16 (10.1)	38 (9.9)	>0.99
30-day echocardiography, *n* = 411				
Mean transvalvular gradient (mmHg)	9.9 (5.1)	7.4 (3.2)	11.1 (5.4)	<0.001
Aortic regurgitation grade				
None/trace	138 (33.6)	21 (16.5)	117 (41.2)	
Mild	260 (63.3)	102 (80.3)	158 (55.6)	
Moderate	12 (2.9)	4 (3.1)	8 (2.8)	
Severe	1 (0.2)	0 (0.0)	1 (0.4)	

Values are presented as median (interquartile range), mean ± SD, or absolute number (%). PTA, percutaneous transluminal angioplasty; AR, aortic regurgitation; BMI, body mass index; IMC, intermediate care; ICU, intensive care unit; MI, myocardial infarction.

a Freedom from mortality; successful access, delivery of the device, and retrieval of the delivery system; correct positioning of a single prosthetic heart valve into the proper anatomical location; freedom from surgery or intervention related to the device or to a major vascular or access-related, or cardiac structural complication (at exit from the procedure room).

b Conversion to surgery was necessary in one patient in the SAPIEN 3 group due to Type A aortic dissection. Annulus rupture (*n* = 2), aortic dissection (*n* = 2), pericardial tamponade (*n* = 3), and access site intervention (*n* = 3) accounted for the remaining complications.

c Technical success, freedom from mortality, freedom from surgery or intervention related to the device or to a major vascular or access-related or cardiac structural complication, and intended performance of the valve.

d Mean gradient <20 mmHg, peak velocity <3 m/s, Doppler velocity index ≥0.25, and less than moderate aortic regurgitation.

e Moderate-to-severe patient prosthesis mismatch=indexed effective orifice area ≤0.85 cm^2^/m^2^ for non-obese patients (BMI < 30 kg/m^2^) or ≤0.70 cm^2^/m^2^ for obese patients (BMI ≥ 30 kg/m^2^).

f Requiring thrombin injection, percutaneous angioplasty, embolectomy, or surgery.

g Subset without pre-existing pacemaker (*n* = 544).

All-cause in-hospital mortality was 2% in the ACURATE and SAPIEN 3 groups (*p* > 0.99), and fatal and disabling stroke also occurred in 2% in both the groups (*p* = 0.95). The incidence of new PPI in pacemaker-naive patients was 10% in both the ACURATE and SAPIEN 3 groups (*p* > 0.99).

**Figure 4:**
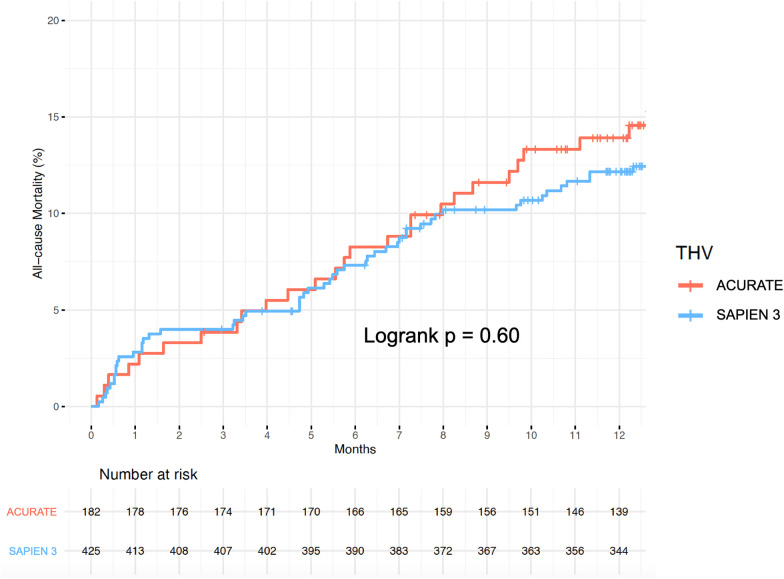
Kaplan–Meier estimate of 1-year all-cause mortality. THV, transcatheter heart valve.

The estimated mortality at 1 year was 13.9% vs. 12.2% (log-rank *p* = 0.60) in the ACURATE and SAPIEN 3 groups, with 7 (4%) and 17 (4%) patients censored, respectively (Figure 4).

### 3.1 Small annulus subgroup

The selected baseline clinical characteristics and postprocedural in-hospital outcomes for patients with a small annulus (*n* = 178) are shown in Table 4. The subgroup comprised older (median age 83 years), predominantly female (80%) patients. An ACURATE device was used in 52% (92/178) of patients, with SAPIEN used in 48% (86/178). Overall, the VARC-3 intended performance rates were 96% and 87% (*p* = 0.064) in the ACURATE and SAPIEN 3 groups, respectively. Moderate or greater AR occurred in 2% and 0% (*p* = 0.50) and mean transvalvular gradient ≥20 mmHg in 1% and 9% (*p* = 0.032) in the ACURATE and SAPIEN 3 groups, respectively.

**Table 4:** Selected baseline clinical characteristics, procedural characteristics, and in-hospital outcomes for patients with small annulus (annulus area <430 mm^2^).

Variable	Total *n* = 178	ACURATE *n* = 92	SAPIEN 3 *n* = 86	*p* value
Clinical characteristics				
Women	142 (79.8)	83 (90.2)	59 (68.6)	0.001
Age (years)	83 (78–87)	84 (79–88)	82 (77–86)	0.056
EuroSCORE II (%)	5.2 (3.3–9.1)	5.7 (3.3–9.7)	4.7 (3.1–7.8)	0.23
LVEF (%)	60 (54–64)	59 (54–63)	61 (55–65)	0.25
Impaired LV function (LVEF <30%)	8 (4.5)	2 (2.2)	6 (7.0)	0.24
Procedural characteristics				
Intended performance^a^	150 (92.0)	81 (96.4)	69 (87.3)	0.064
AR grade ≥ II	2 (1.1)	2 (2.2)	0 (0.0)	0.497
Elevated gradient (≥20 mmHg)	9 (5.1)	1 (1.1)	8 (9.4)	0.032
Mean transvalvular gradient (mmHg)	10.7 ± 4.8	8.2 ± 3.3	13.3 ± 4.7	<0.001
Patient prosthesis mismatch^b^	52 (31.9)	15 (17.9)	37 (46.8)	<0.001
In-hospital outcomes				
All-cause mortality	2 (1.1)	1 (1.1)	1 (1.2)	>0.99
Major vascular complication	18 (10.1)	11 (12.0)	7 (8.1)	0.55
Annulus rupture	2 (1.1)	0 (0.0)	2 (2.3)	0.45
Access site complication requiring intervention^c^	8 (4.5)	6 (6.5)	2 (2.3)	0.32
New pacemaker^d^	9 (5.5)	5 (6.0)	4 (4.9)	>0.99

Values are presented as median (interquartile range), mean ± SD, or absolute number (%). AR, aortic regurgitation; BMI, body mass index; LVEF, left ventricular function.

a Mean gradient <20 mmHg, peak velocity <3 m/s, Doppler velocity index ≥0.25, and less than moderate aortic regurgitation.

b Moderate-to-severe patient prosthesis mismatch = indexed effective orifice area ≤0.85 cm^2^/m^2^ for non-obese patients (BMI < 30 kg/m^2^) or ≤0.70 cm^2^/m^2^ for obese patients (BMI ≥ 30 kg/m^2^).

c Requiring thrombin injection, percutaneous angioplasty, embolectomy, or surgery.

d Subset without pre-existing pacemaker (*n* = 165).

### 3.2 Univariate and multivariate analysis

The results of the linear regression analysis to evaluate the association between relevant factors and postprocedural iEOA are shown in Table 5. In multivariate modelling, LVEF <30% and a smaller annulus area were significantly associated with a smaller postprocedural indexed orifice area. The use of the ACURATE device was associated with significantly larger iEOA.

**Table 5:** Linear regression analysis evaluating the association between relevant variables and postprocedural indexed orifice area.

Variable	Univariate			Multivariate Model adjusted R^2^ = 0.07	
	*β*	R^2^	*p*	*β*	*p*
Categorical variables					
Women	−0.067	0.011	0.011	−0.062	0.050
LVEF < 30%	−0.171	0.021	<0.001	−0.189	<0.001
Predilatation	0.070	0.012	0.009	0.028	0.36
Postdilatation	0.020	0.001	0.54		
Self-expanding THV	0.091	0.018	0.001	0.131	<0.001
Continuous variables					
Age (years)	0.003	0.004	0.123		
EuroSCORE II	−0.001	0.001	0.49		
Annulus area (mmHg)	0.000	0.009	0.023	0.001	0.004
Agatston score	0.000	0.000	0.86		

Unadjusted and adjusted associations of variables were related to postprocedural indexed effective orifice area using linear regression modelling. The multivariate model included 566/607 patients. β is the size of the effect on the indexed effective orifice area (cm^2^/m^2^) vs. the reference category (categorical), or per unit increase in the independent variable (continuous). Variables with a univariate *p* < 0.05 were included in the multivariate model. LVEF, left ventricular ejection fraction; THV, transcatheter heart valve.

## 4 Discussion

This study demonstrates that a prospective valve selection strategy partnering the ACURATE neo/neo2 and SAPIEN 3/3 Ultra THVs can achieve high and comparable overall valve performance in a broad population of patients with severe native valve AS. To the best of our knowledge, this is the first study to investigate a prospective selection strategy for balloon-expandable versus self-expanding THV. Both devices were associated with high VARC-3 technical success and intended valve performance (Central Illustration). Intended performance was significantly higher in the ACURATE group because of the lower transvalvular gradients. At discharge, all-cause mortality, fatal and disabling stroke, and new PPI were comparable between the groups. There was no significant difference in the estimated 1-year mortality rate.

**CENTRAL ILLUSTRATION:**
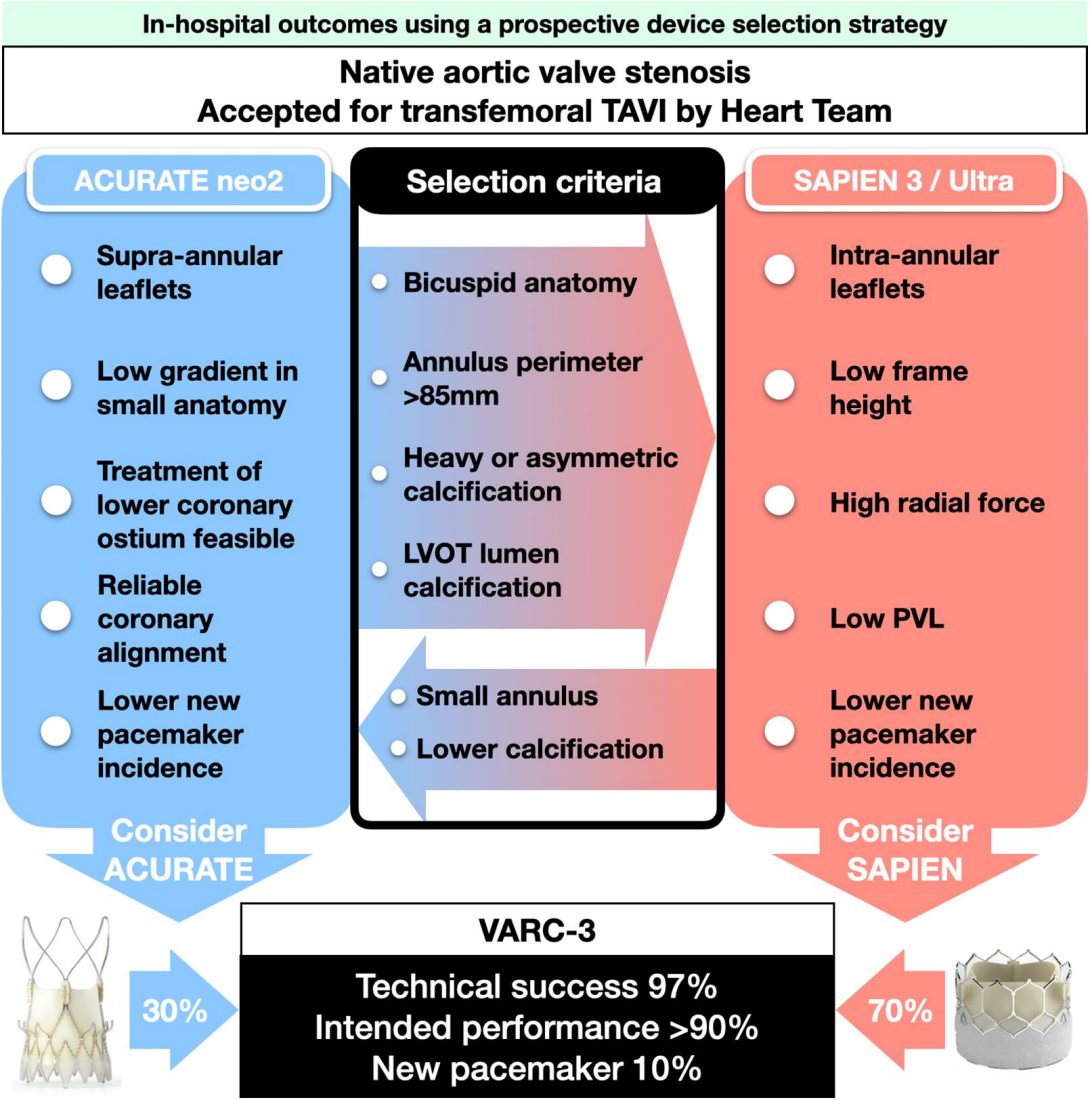
Diagram showing relevant technical features of the self-expanding ACURATE and balloon-expandable SAPIEN devices, aortic valve selection criteria employed in this study, and a summary of in-hospital outcomes. The ACURATE device can treat an upper annulus perimeter of 85 mm; larger annuli were treated with SAPIEN 3.

### 4.1 TAVI device selection

The proliferation of TAVI devices available for the treatment of AS has increased the options available to physicians. Existing data indicate that SEVs are typically associated with a lower transvalvular gradient at the expense of higher new PPI and PVL (4, 14, 15). Available randomised controlled trials have often excluded patients with bicuspid aortic valves, and inclusion bias due to improving operator experience may have occurred during patient enrolment, limiting the generalisability of randomised trial results to the broader TAVI population. We hypothesised that haemodynamic outcomes could be optimised in a broad TAVI cohort by careful anatomical selection using a two-valve strategy with the ACURATE SEV and SAPIEN 3 BEV platforms. Although the SAPIEN 3 device was preferred for bicuspid, heavily or asymmetrically calcified anatomy, LVOT lumen calcification, and younger age, patients with lower valve calcification and smaller annulus dimensions were preferentially treated with the ACURATE device. As a result of this selective strategy, the in-hospital overall haemodynamic performance and clinical outcomes were similar between the groups, with significantly higher VARC-3 intended performance in the ACURATE group due to lower transvalvular gradients and less moderate-to-severe patient prosthesis mismatch.

### 4.2 Differences in the baseline and procedural characteristics

In this study, patients selected for treatment with ACURATE were older, with a higher proportion of women, lower mean aortic valve gradient, and lower Agatston score, likely reflecting the smaller annulus dimensions and higher incidence of paradoxical low-flow low-gradient AS with lower aortic valve calcification observed in women (16). During the procedure, predilatation was performed in all ACURATE patients, and postdilatation was employed significantly more frequently than in the SAPIEN 3 group to facilitate optimal THV expansion. The procedure and fluoroscopy times and contrast agent volume were significantly higher in the ACURATE group, likely reflecting the differences in the deployment technique for the two devices.

Notably, although patients treated with ACURATE had higher rates of arterial disease (combined peripheral, carotid, and aortic), the preference for the SAPIEN 3 device for more challenging access anatomy is demonstrated by the higher rate of super-stiff wire use for insertion of the primary sheath (Boston Scientific Meier wire 24% vs. 30%, respectively).

### 4.3 Clinical outcomes

The rate of clinical outcomes in this study compared favourably with existing data. In-hospital all-cause mortality in this study was 2% in the ACURATE and SAPIEN 3 groups, respectively, compared with 3% and 2% thirty-day mortality reported in the meta-analysis data (15). The incidence of 1-year mortality in our all-comer cohort was comparable to the 12% rate observed in the TAVI arm of the PARTNER 2 study of intermediate surgical-risk patients (7).

Two patients in the SAPIEN 3 group experienced partial annulus rupture. In one case, LVOT calcification was absent, and rupture may have been related to asymmetric, moderate-to-severe leaflet calcification. In the other case, mild LVOT calcification (22 mm^3^) and moderate-to-severe annulus calcification with excessive oversizing of the THV may have been implicated. Both patients were successfully treated with conservative management after pericardial drainage. There were no episodes of annulus rupture in patients with ≥moderate LVOT calcification or in the ACURATE group. The low incidence of annulus rupture and the relatively small sample size in this study preclude drawing further conclusions regarding differences in annulus rupture rates between groups, although this complication is known to occur more frequently in BEV (3).

All in-hospital strokes occurred in 4% of ACURATE and SAPIEN 3 patients, compared with the 2%–3% thirty-day stroke rate using the ACURATE neo SEV in the randomised SCOPE I and II studies and 1% using the ACURATE neo2 SEV in the ACURATE IDE trial (5, 6, 9). In SCOPE I, a 30-day stroke rate of 3% was observed in patients receiving a SAPIEN 3 device, whereas a rate of <1% was observed in low-risk patients in the PARTNER 3 study and in the SAPIEN 3 arm of the ACURATE IDE trial (5, 8, 9). In our study, all in-hospital stroke events were assessed by a neurologist and evaluated using neuroimaging when clinically indicated, which may have influenced the detection of stroke events compared with published data.

The incidence of residual AR, generally because of PVL, is consistently higher in SEV than in BEV (15) and is associated with worse clinical outcomes (17). In our study, in-hospital mild AR was more likely to occur in the ACURATE group. The 2% incidence of in-hospital ≥moderate AR with the ACURATE SEV platform in this study was markedly lower than the 9%–10% rate observed in the randomised SCOPE I and II trials using the first-generation ACURATE neo device and is comparable with the in-hospital 1% rate observed using the ACURATE neo2 device in the ACURATE IDE trial (5, 6, 9). The lower AR rate with the newer ACURATE neo2 THV potentially reflects the impact of the sealing skirt and/or improved anatomical selection with increasing operator experience. In the current study, no patients receiving the older ACURATE neo device had ≥moderate AR, suggesting that careful anatomical selection with the ACURATE neo/neo2 device may be the determining factor in reducing PVL. Despite the anticipated negative impact of residual AR, it should be noted that a composite of all-cause death, stroke, and rehospitalisation for heart failure between patients randomised to an ACURATE neo or SAPIEN 3 device in the SCOPE I study was not significantly different at the 3-year follow-up (18). Reported AR rates at 30 days with the SAPIEN 3 BEV platform are known to be lower than those with SEV (15), with a 30-day ≥moderate AR rate of <1% observed in the ACURATE IDE study using the SAPIEN 3 platform (9). Furthermore, in the present study, the elevated mean transvalvular gradient ≥20 mmHg was lower in the ACURATE group (<1% vs. 4%). Although SEVs generally exhibit lower transvalvular gradients (15), as observed in this study, the 4% rate of mean postprocedural gradient ≥20 mmHg in the SAPIEN 3 group compared favourably with comparable registry data, suggesting a rate of 8% in BEVs (19). The very low rate of ≥moderate AR and lower rate of elevated transvalvular gradient ≥20 mmHg in the SAPIEN 3 group in this analysis may also reflect the high proportion of SAPIEN 3 Ultra devices used, which are associated with improved THV haemodynamic function (20). The stepwise deployment strategy employed to optimise BEV expansion may have also impacted the valve haemodynamic function in this study (12).

Moderate-to-severe patient prosthesis mismatch using the VARC-3 definition was significantly lower in the ACURATE cohort than in SAPIEN 3 in the current analysis. This finding was accentuated in the small annulus subgroup, consistent with the results of the randomised SMART study (4). However, the clinical significance of an elevated gradient in BEV remains uncertain, with no impact on mortality observed at 5-year follow-up in the randomised PARTNER 2 study using the SAPIEN XT THV in patients with small annuli (data presented by Dr. Hahn at New York Valves 2024), nor in published registry data (21, 22). In multivariate linear regression analysis, the use of an ACURATE device was significantly associated with a larger postprocedural iEOA, whereas LVEF <30% and a smaller native aortic valve annulus area were associated with a smaller postprocedural iEOA. No significant association was found between predilatation, postdilatation, age, EuroSCORE II, Agatston score, and postprocedural iEOA.

In-hospital new PPI in this study was 10% in both groups, with no significant difference between ACURATE and SAPIEN 3, comparable with existing data (15). Furthermore, no significant differences were observed in major vascular complications or access site complications requiring intervention.

### 4.4 Combining ACURATE neo2 and SAPIEN 3/ultra

The medical reimbursement system in many European countries has precipitated cost-saving measures in hospitals performing TAVI. This study demonstrates that combining the ACURATE neo2 and SAPIEN 3/Ultra THVs can reduce the dependency on a single device without compromising clinical outcomes in an all-comer group of patients with native valve AS. In this study, 30% of the patients received an ACURATE THV. The larger ACURATE Prime XL™ valve was not available at the time of this study.

### 4.5 Limitations

Shortly before completion of this analysis, Boston Scientific withdrew the ACURATE platform from the worldwide market; however, the selective device strategy employed in this study may be applicable to other valve types and may be useful if the ACURATE or a similar device returns to the market.

Device selection in this single-centre study was determined by the implanting operators according to predefined qualitative anatomic features. Although the authors attempted to define criteria to assist in device selection, confounding factors may have impacted the final decision in some cases. Although the definition of challenging vascular access in the existing literature is inconsistent, in our study, these patients were identified based on operator experience and treated preferentially with the SAPIEN 3 device; however, minimal vessel diameter, calcification severity, and tortuosity were not formally evaluated. The non-randomised nature of this device selection strategy study indicates that the findings of this single-centre analysis may not be generalisable to the wider TAVI population. Further studies with randomisation to a defined “device selection strategy” versus “standard care” are necessary to definitively confirm any advantages of targeted device selection. However, the pragmatic implications of our study may be immediately applicable to centres with a limited range of available THV types.

A small percentage of patients during the study period were treated with alternative THVs, typically when a retrievable device was deemed essential for the safety of the procedure. Furthermore, the more complex device crimping process for the ACURATE THV necessitated increased company support and may have resulted in some patients in this study receiving SAPIEN 3 for purely logistical reasons.

Although mortality data at 1-year were available for virtually all patients, incomplete follow-up prevented the evaluation of other relevant clinical endpoints. The lack of centralised patient follow-up hampered the collection of many relevant clinical endpoints, including 30-day echocardiography results. Finally, the latest-generation SAPIEN 3 ULTRA Resilia device did not have regulatory approval in Europe and was not available for this study.

## 5 Conclusion

In a broad population of patients undergoing transfemoral TAVI for native valve AS, a prospective THV selection strategy using the ACURATE neo/neo2 SEV and SAPIEN 3/SAPIEN 3 Ultra BEV demonstrated high VARC-3 technical success and intended performance, with low and comparable in-hospital clinical adverse events. All-cause mortality at 1 year did not differ significantly between the devices. To the best of our knowledge, this is the first study to prospectively select SEV versus BEV based on the technical features of the device. This study suggests that it is possible to compensate for the limitations of existing TAVI valves by using a patient-specific device selection strategy.

## Data Availability

The data analysed in this study are subject to the following licenses/restrictions: Swiss TAVI Registry data. Requests for access to these datasets should be directed to jonathan.michel@usz.ch.
